# Similarity of aberrant DNA methylation in Barrett's esophagus and esophageal adenocarcinoma

**DOI:** 10.1186/1476-4598-7-75

**Published:** 2008-10-02

**Authors:** Eric Smith, Neville J De Young, Sandra J Pavey, Nicholas K Hayward, Derek J Nancarrow, David C Whiteman, B Mark Smithers, Andrew R Ruszkiewicz, Andrew D Clouston, David C Gotley, Peter G Devitt, Glyn G Jamieson, Paul A Drew

**Affiliations:** 1 Discipline of Surgery, School of Medicine, The University of Adelaide, Royal Adelaide Hospital, Adelaide, South Australia, 5005, Australia; 2 Oncogenomics, Queensland Institute of Medical Research, Brisbane, 4006, Australia; 3 Division of Population Studies and Human Genetics, Queensland Institute of Medical Research, Brisbane, 4006, Australia; 4 School of Medicine, University of Queensland, Brisbane, Queensland, 4072, Australia; 5 Division of Tissue Pathology, Institute of Medical and Veterinary Science, Adelaide, South Australia, 5000, Australia; 6 School of Nursing and Midwifery, Flinders University, Bedford Park, South Australia, 5042, Australia

## Abstract

**Background:**

Barrett's esophagus (BE) is the metaplastic replacement of squamous with columnar epithelium in the esophagus, as a result of reflux. It is the major risk factor for the development of esophageal adenocarcinoma (EAC). Methylation of CpG dinucleotides of normally unmethylated genes is associated with silencing of their expression, and is common in EAC. This study was designed to determine at what stage, in the progression from BE to EAC, methylation of key genes occurs.

**Results:**

We examined nine genes (APC, CDKN2A, ID4, MGMT, RBP1, RUNX3, SFRP1, TIMP3, and TMEFF2), frequently methylated in multiple cancer types, in a panel of squamous (19 biopsies from patients without BE or EAC, 16 from patients with BE, 21 from patients with EAC), BE (40 metaplastic, seven high grade dysplastic) and 37 EAC tissues. The methylation frequency, the percentage of samples that had any extent of methylation, for each of the nine genes in the EAC (95%, 59%, 76%, 57%, 70%, 73%, 95%, 74% and 83% respectively) was significantly higher than in any of the squamous groups. The methylation frequency for each of the nine genes in the metaplastic BE (95%, 28%, 78%, 48%, 58%, 48%, 93%, 88% and 75% respectively) was significantly higher than in the squamous samples except for CDKN2A and RBP1. The methylation frequency did not differ between BE and EAC samples, except for CDKN2A and RUNX3 which were significantly higher in EAC. The methylation extent was an estimate of both the number of methylated alleles and the density of methylation on these alleles. This was significantly greater in EAC than in metaplastic BE for all genes except APC, MGMT and TIMP3. There was no significant difference in methylation extent for any gene between high grade dysplastic BE and EAC.

**Conclusion:**

We found significant methylation in metaplastic BE, which for seven of the nine genes studied did not differ in frequency from that found in EAC. This is also the first report of gene silencing by methylation of ID4 in BE or EAC. This study suggests that metaplastic BE is a highly abnormal tissue, more similar to cancer tissue than to normal epithelium.

## Background

The incidence of esophageal adenocarcinoma (EAC) is increasing rapidly and patient outcomes remain poor. Known risk factors for EAC include obesity, gastro-esophageal reflux, and the presence of Barrett's esophagus (BE). Repeated injury from gastro-duodenal reflux is thought to result in the replacement of the esophageal squamous mucosa with a metaplastic columnar lined epithelium. The presence of goblet cells within columnar epithelium is diagnostic for BE. Approximately 0.5 – 1% of patients with BE will develop EAC each year and patients with BE have a 50- to 100-fold increased risk of EAC compared to the general population [1,2].

The progression from BE to EAC is generally accepted to proceed via the histological stages of low-grade and high-grade dysplasia. Progress along this pathway appears to mirror the accumulation of genetic abnormalities, with a number of reports suggesting a stepwise progression of genetic changes. Abnormalities in CDKN2A, seen in BE metaplastic tissue, followed by altered TP53 expression, generally reported in dysplastic tissue, have been associated with the transition from BE to EAC [3,4]. Alterations in the expression of many other genes have also been described at different stages of the progression to cancer [5,6].

Genes can be aberrantly down-regulated as a result of genomic alterations such as mutation, deletion, or DNA methylation. In humans, methylation of cytosines generally occurs in the context of a CpG dinucleotide. Regions of relatively high CpG content, termed CpG islands, are found in the promoter region of many genes. Methylation within these regions is associated with suppression of the expression of certain genes, for example the switching off of key developmental genes in adult tissues. However, aberrant methylation of normally unmethylated CpG islands is a common cause of altered gene expression in cancer. As esophageal biopsies are easily obtained at endoscopy, it is possible to determine at what stage methylation occurs during the progression to cancer. In this study we compared the methylation of nine genes, frequently methylated in other cancers, between squamous, BE and EAC tissues.

## Results

### Gene methylation and expression in esophageal cancer cell lines

**Figure 1:**
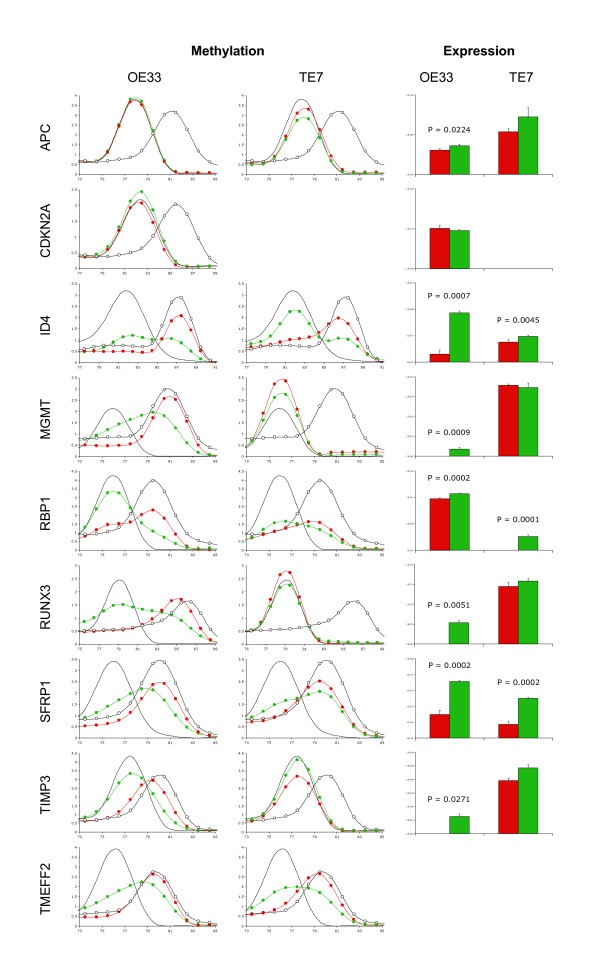
**Methylation and expression of APC, CDKN2A, ID4, MGMT, RBP1, RUNX3, SFRP1, TIMP3 and TMEFF2 in esophageal cancer cell lines OE33 and TE7**. The esophageal cancer cell lines OE33 and TE7 were treated with either 1 μmol/L aza-dC or vehicle for 72 hours. The medium was replaced with fresh medium only, and the cells incubated for a further 24 hours before harvesting. Bisulphite modified DNA was amplified using primers and PCR conditions (Table 2) which were specific for bisulphite modified DNA, did not discriminate between methylated and unmethylated sequences, and did not amplify unmodified DNA. The PCR products were melted by increasing the temperature from 60 to 95°C, rising 0.5 or 1°C at each step, waiting 30 seconds on the first step then 5 seconds for each step thereafter. Data was collected and analysed using the Melt Curve Analysis function of the RG-3000 Application Software. The left hand column shows the melt curves for each of the nine genes for OE33, the central column for TE7. Each plot shows the melt curves for the unmethylated (black lines) and methylated (open circles) controls and the cell lines treated with vehicle (red circles) or aza-dC (green circles). The horizontal axis represents temperature and the vertical axis -dF/dT. CDKN2A was not amplified in TE7. Interpretation of the melt curves is described in the Materials and Methods. The right hand column shows the gene expression in cell lines treated with vehicle (red columns) or aza-dC (green columns), as determined by qRT-PCR and normalised to HMBS. Data shown are the means ± SD from three independent experiments. TMEFF2 expression was below detectable limits in either cell line treated with vehicle or aza-dC.

Methylation and expression of the nine genes listed in Table 1 were measured in triplicate cultures of the esophageal cancer cell lines OE33 and TE7, following treatment with either 5-aza-2'-deoxycytidine (aza-dC) or vehicle (Figure 1). Methylation was observed in all genes in OE33, except APC and CDKN2A, while in TE7 it was only observed in ID4, RBP1, SFRP1, and TMEFF2. Treatment with aza-dC reduced methylation and significantly increased the expression of all methylated genes except TMEFF2, which was undetected in either cell line. CDKN2A was not detected in TE7 by methylation PCR or quantitative real-time reverse-transcription PCR (qRT-PCR), suggesting that the gene was deleted in this cell line. Following treatment with aza-dC, transcript levels were not significantly increased for any unmethylated genes, except for APC in OE33.

**Table 1:** The primer sequences and annealing temperatures for methylation analysis

Gene	Forward primer	Reverse primer	Annealing temperature (°C)
APC	GAAGYGGAGAGAGAAGTAGTTG	ACRAACTACACCAATACAACCACATA	55
CDKN2A	TYGGYGGYGGGGAGTAGTATGGAGTTT	RTTAAACAACRCCCCCRCCTCCAACAA	60
ID4	GGGGYGTAYGGTTTTATAAATATAGTTG	TAATCACTCCCTTCRAAACTCCGACTAAA	55
MGMT	5G5GTTT5GGATATGTTGGGATAGTT	AC5AAAC5ACCCAAACACTCACCAAA	55
RBP1	TGTGYGYGTTGGGAATTTAGTTG	CRAAAAATAACTAAAACCAATTAACCACAAA	55
RUNX3	YGTYGTTTTTTGYGTTTTGAGGTT	ACTTAAATCTACRAAAATACRCATAACAA	55
SFRP1	ATTTTYGGGAGTYGGGGYGTATT	RACCAATAACRACCCTCRACCTA	57
TIMP3	TTTGAGGGGGYGGGTTTTAATAGTT	AACRACCTCCCRACGAAAAAACAAA	55
TMEFF2	TTGTTTTTTYGTYGGGTGTTATTGTTAT	AACAAACRACTTCCRAAAAACACAAA	55

### Methylation in esophageal adenocarcinoma and Barrett's esophagus

**Figure 2:**
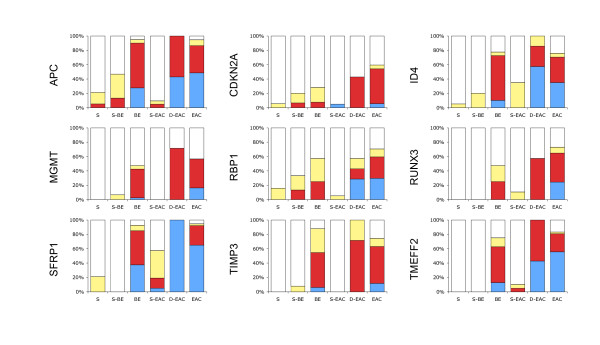
**Methylation frequency and methylation extent in esophageal tissues**. Methylation of each gene was measured in multiple biopsies of squamous mucosa (S, n = 19) from seven patients without BE, single biopsies of squamous mucosa (S-BE, n = 16) and multiple biopsies of columnar mucosa (BE, n = 40) from 18 patients with BE, single biopsies of squamous mucosa (S-EAC, n = 21), high grade dysplastic Barrett's (D-EAC, n = 7) and tumor (EAC, n = 37) from 38 patients with EAC. The methylation was graded as unmethylated (white), methylated 1 (yellow), 2 (red), or 3 (blue), as described in the Materials and Methods.

The results in Figure 2 show the methylation frequency, the percentage of samples that had any extent of methylation, for each of the nine genes, in squamous tissues from either patients with no known history of BE (S), or patients with BE (S-BE) or patients with adenocarcinoma (S-EAC), in metaplastic Barrett's from patients with BE but no dysplasia or adenocarcinoma (BE), in high grade dysplastic Barrett's from patients with adenocarcinoma (D-EAC), and in adenocarcinoma (EAC). In squamous tissues the methylation frequency for each gene did not differ significantly between S and S-BE, while in S-EAC methylation was significantly higher for SFRP1 (compared to either S or S-BE) and ID4 (compared to S) and lower for APC (compared to S-BE). For all nine genes the methylation frequency in BE and D-EAC was significantly higher than each of the squamous tissues (S, S-BE and S-EAC) with the exception of CDKN2A (no difference between BE or D-EAC and S or S-BE), RBP1 (BE vs S-BE and D-EAC vs S or S-BE) and SFRP1 (D-EAC vs S-EAC). There were no differences between BE and D-EAC. The methylation frequency for all nine genes was significantly higher in EAC than any squamous tissue. There were no differences between EAC and D-EAC. There were no differences between EAC and BE, except for CDKN2A and RUNX3, which were significantly higher in EAC than BE (P = 0.0104 and 0.0358 respectively).

The methylation extent, which reflects the combination of both the number of methylated alleles and the density of methylation of those alleles, was graded on a scale of 1–3, with 1 being low and 3 being high methylation (Figure 2). The methylation extent in EAC was significantly greater than in BE for all genes, except for APC, MGMT and TIMP3. There were no significant differences in methylation extent between EAC and D-EAC.

**Figure 3:**
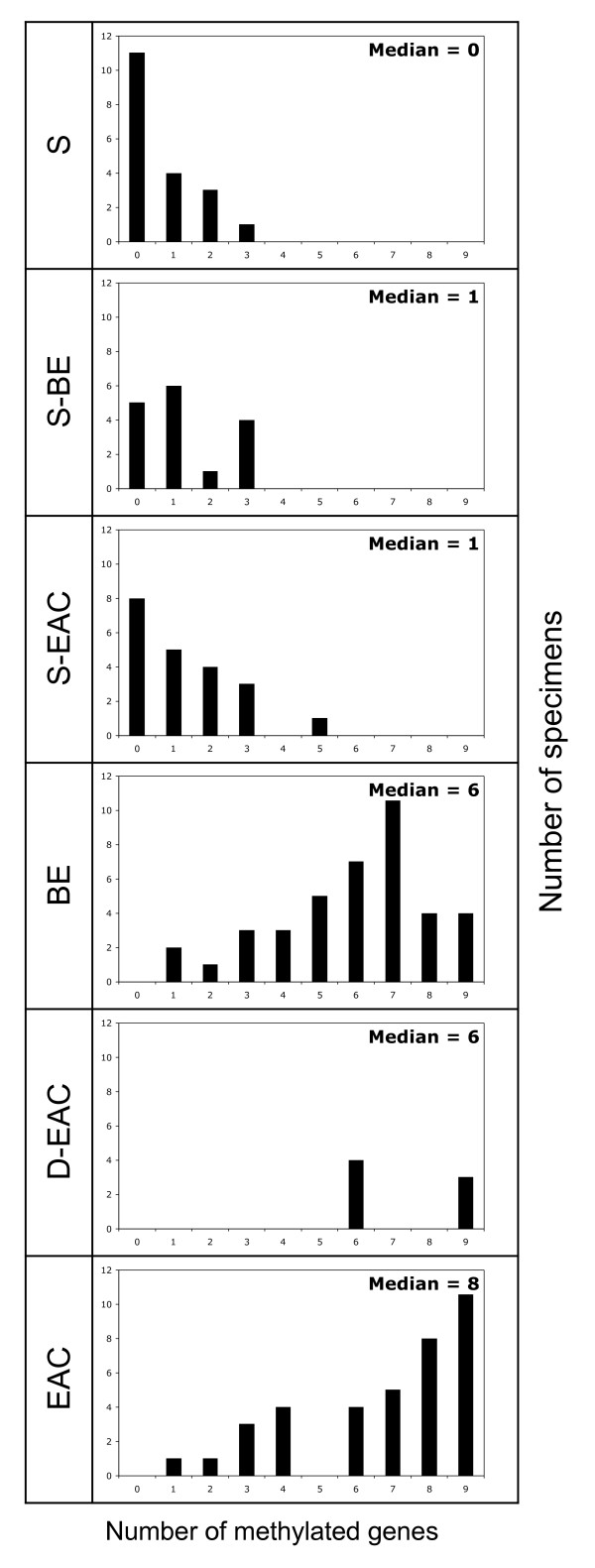
**Number of methylated genes in esophageal tissues**. The number of methylated genes in specimens of squamous mucosa from patients without BE (S), squamous mucosa (S-BE) and columnar mucosa (BE) from patients with BE, and squamous mucosa (S-EAC), high grade dysplastic Barrett's (D-EAC) and adenocarcinoma (EAC) from 38 patients with EAC.

The number of genes methylated in each specimen is shown in Figure 3. Significantly more genes were methylated in specimens of BE, D-EAC and EAC, compared to any of the squamous tissues (P < 0.01 for each comparison). There were no significant differences between any of the squamous tissues, nor between BE, D-EAC and EAC.

### Methylation and gene expression in esophageal tissues

**Figure 4:**
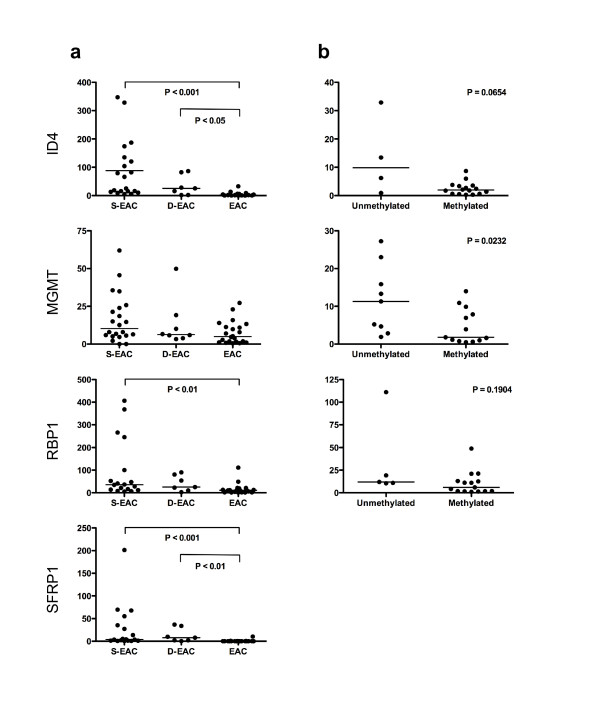
**Expression and methylation in esophageal tissues**. (a) Normalised mRNA expression of ID4, MGMT, RBP1, and SFRP1 in squamous (S-EAC), high grade dysplastic Barrett's (D-EAC) or tumor (EAC) tissues from patients with EAC. (b) Normalised expression of ID4, MGMT, and RBP1 in tumors unmethylated or methylated for the gene. The horizontal bar represents the median expression.

We examined the relationship between methylation and gene expression in tissues from patients with EAC, from which sufficient RNA was available (Figure 4). The expression of APC, CDKN2A, MGMT, RUNX3, TIMP3 and TMEFF2 did not differ significantly between EAC, D-EAC and S-EAC. There was significantly less expression of ID4, RBP1 and SFRP1 in EAC compared to S-EAC, and of ID4 and SFRP1 in EAC compared to D-EAC (Figure. 4a).

We then looked for an association between the presence of methylation and a reduction in gene expression within the EAC tissues. There was no significant difference in APC, CDKN2A, ID4, RBP1, RUNX3 and TIMP3 expression levels between methylated and unmethylated tumors. There were too few SFRP1 unmethylated tissues for this analysis. There was significantly less expression of MGMT in methylated tumors compared to unmethylated tumors (Figure. 4b). Furthermore, the expression of MGMT did not differ between unmethylated squamous and unmethylated tumor tissues, suggesting that the observed reduction in EAC was primarily due to methylation, not phenotypic differences.

## Discussion

Methylation is an important mechanism for the silencing of genes in the development of cancer. We compared the frequency and extent of methylation of APC, CDKN2A, ID4, MGMT, RBP1, RUNX3, SFRP1, TIMP3, and TMEFF2 in esophageal adenocarcinoma (EAC), high grade dysplastic Barrett's from patients with adenocarcinoma (D-EAC), metaplastic Barrett's from patients without dysplasia or adenocarcinoma (BE), and histologically normal esophageal squamous epithelium. All nine genes showed an increase in frequency of methylation in EAC compared to squamous epithelium. We found that for seven of these genes there was no difference in the frequency of methylation between the BE and EAC. For only CDKN2A and RUNX3 was there a significant increase in frequency of methylation in EAC compared to BE. The extent of methylation in EAC was significantly greater than in BE for six of the nine genes (CDKN2A, ID4, RBP1, RUNX3, SFRP1 and TMEFF2) but was not different to D-EAC for any gene. This suggests that methylation of these genes can occur early in the development of Barrett's metaplasia, and for only some of these genes does the extent of methylation increases during the progression to cancer.

In the technique we used to analyse methylation, bisulphite modified DNA is amplified using primers and conditions that do not discriminate between methylated and unmethylated sequences. Amplification is therefore independent of the presence or extent of methylation within the region being assessed. Unlike two common methods for measuring methylation, Methylation-Specific PCR [7] or MethyLight [8], the method we used will detect small amounts of methylation anywhere along the target sequence. Thus the methylation frequency we measure may differ from that reported by others. The methylation extent reflects a combination of the percentage of the alleles that are methylated, and the density of methylation on each allele. To determine if there was a relationship between the methylation measured in our assays and transcriptional silencing we measured gene expression in esophageal cell lines treated with the demethylating drug aza-dC. There was an increase in expression of ID4, MGMT, RBP1, RUNX3, SFRP1 and TIMP3, suggesting an association between methylation and silencing of expression for these genes. A relationship could not be determined for APC or CDKN2A, which were unmethylated, or TMEFF2, for which we could not measure any transcript in either cell line. For these three genes we have shown using cell lines established from other cancers that methylation of the regions we amplified are associated with gene silencing (unpublished observations). In our EAC tissues only methylation of MGMT was associated with a significant reduction in mRNA expression. The lack of a significant difference in gene expression for the other genes may be due to methylation of only one allele, or the presence of unmethylated cells in the tissue, such as clonal variants of the tumor, or stromal or infiltrating cells. Methylation in the cancer tissues was always greater than in the squamous epithelium of patients without cancer, so even if methylation did not result in gene silencing, it was still associated with cancer.

The processes involved in the transition from squamous epithelium to Barrett's metaplasia are unknown, although chronic gastro-esophageal reflux is widely believed to be the main trigger, with approximately 10% of gastro-esophageal reflux patients having BE [9]. It is accepted that BE is a pre-malignant condition, which over time, as a result of the accumulation of multiple genetic and epigenetic modifications, can progress through dysplasia to EAC in a percentage of patients. A large number of such molecular changes have been reported in BE and EAC, although the sequence of these changes appear not to be as predictable as in some other cancers such as in the colon [6].

It is not clear from the literature when aberrant DNA methylation occurs during the progression to EAC. There may be differences in molecular profile of BE tissue obtained from cancer resection specimens, compared to BE tissue obtained from patients with metaplasia only. Our samples of BE were obtained from patients with no detectable dysplasia or cancer, and were compared to cancer tissue from different patients. High levels of methylation in such BE specimens have been reported by others, such as methylation of SFRP1 being as frequent in BE (81 – 100% of samples) as it is in EAC (93 – 100%) [10,11]. In several studies high levels of methylation have been found in BE for many genes, but a few have reported low methylation in BE and then high in EAC tissues. Eads et al examined a single biopsy of metaplastic tissue from six patients with BE without associated EAC and reported methylation of APC, MGMT and TIMP3 in BE and also in cancers from other patients, but they did not compare the amounts of methylation in each [12]. They also reported four genes, CDKN2A, ESR1 and MYOD1 and CALCA as unmethylated in these BE patients, and methylated in the cancers. Schulmann et al, using MethyLight, measured methylation in 93 biopsies of BE from an undisclosed number of patients [13]. Methylation was as frequent in samples of EAC (n = 77) as in BE for APC, MGMT, RBP1 and TIMP3, but was significantly greater in EAC compared to BE for CDKN2A, RUNX3 and TMEFF2. Furthermore, the methylation level, their measure of the number of extensively methylated or hypermethylated molecules in the specimen, increased significantly from BE to dysplasia for these three genes. Clement reported that APC, TIMP3 and TERT were significantly more methylated in BE of patients who progressed to EAC compared to patients with BE which had not progressed during a follow-up of between four to ten years [10]. Of the nine genes which we studied, only methylation of CDKN2A and RUNX3 appeared to increase in frequency in the progression from BE to cancer, and only six of the nine genes increased in the extent of methylation.

The underlying mechanisms that cause aberrant DNA methylation in Barrett's metaplasia and cancer are unknown. Our study confirms that aberrant methylation of multiple genes is an early event, and mostly occurs independently of dysplasia or EAC. Thus, we speculate that methylation of some genes occurs at or shortly after the transition from squamous to columnar metaplasia. To date there are no longitudinal studies investigating methylation changes during metaplasia.

This is the first report of ID4 methylation in BE or EAC. The other genes in this study have previously been reported to be methylated in esophageal disease. ID4 is a member of the inhibitor of DNA binding (ID) family of proteins that inhibit the binding of basic helix-loop helix transcription factors to DNA. It regulates the transcription of genes important in development and differentiation, and is a candidate tumor suppressor gene [14,15]. Methylation of ID4 has been reported in and associated with the silencing of gene transcription and loss of protein expression in lymphoma [16], and gastric [17], colorectal [18] and breast carcinoma [19,20]. Methylation of ID4 correlated with increased risk of lymph node metastasis in T1 stage breast cancer [20], and histopathological tumor grade and poorer prognosis in colorectal carcinoma [18]. Our findings that ID4 is frequently methylated in BE and EAC but not in the normal squamous mucosa and that demethylation of cancer cell lines significantly increases expression, suggests loss of ID4 expression is important in the neoplastic progression of BE, supporting its role as a tumor suppressor.

DNA methylation is not the only abnormality reported in the genome of metaplastic Barrett's epithelium of patients without dysplasia or EAC. Using gene expression microarrays, Wang et al reported that the gene expression profile of BE more closely resembled EAC than esophageal squamous epithelium. Rather than BE being a benign tissue, they concluded that it was biologically closer to cancer than to normal squamous epithelium [21]. Genomic loss, chromosomal gains and amplifications, mutations, and aneuploidy are observed in BE [22,23], and loss of heterozygosity of CDKN2A is reported in 47 – 75% of patients with BE, in the absence of dysplasia or EAC [24-26]. In metaplastic epithelium from patients with dysplasia or EAC, 5q (APC), 13q (RB1), 17p (TP53), and 18q (DCC) are commonly lost, whilst 8q, 8p, and 6p are frequently gained [27-29]. In contrast, mutations in BE are relatively uncommon. Mutations in CDKN2A have been reported in up to 7% of patients with metaplasia [24,30], but most reports suggest that mutations are absent in BE from patients without dysplasia or EAC [31].

In this study of methylation in esophageal disease we have measured little methylation in any esophageal squamous epithelium, but in BE tissues there was significant methylation which for all but two of the nine genes examined did not increase in frequency in the progression from BE to EAC. We have also reported methylation of ID4 for the first time in BE or EAC. Together these findings confirm that BE is a precancerous tissue, and that aberrant promoter methylation occurs early in metaplasia before histological evidence of progression towards cancer, and that metaplastic BE is nearly as abnormal epigenetically as EAC.

## Methods

### Patient samples

Single samples of primary esophageal adenocarcinoma (EAC, n = 37), dysplastic Barrett's (D-EAC, n = 7) and histologically normal squamous mucosa from the proximal resection margin (S-EAC, n = 21) from 38 patients with EAC were collected into liquid nitrogen or into RNAlater (Ambion, Austin, TX, USA). Multiple biopsies every 2 cm from within circumferential columnar lined esophagus (BE, n = 40) and a single biopsy of squamous mucosa proximal to the squamo-columnar junction (S-BE, n = 16) from 18 patients with BE were collected into RNAlater. The presence of goblet cells in at least one biopsy from the columnar lined esophagus was confirmed in all patients with BE. Up to three biopsies of squamous mucosa (S, n = 19) from each of seven patients without a known history of BE, but who had undergone a fundoplication for gastro-esophageal reflux disease more than five years earlier, were collected into RNAlater. Clinicopathological details of all patients are summarised in Table 2, and details of each patient are described in Additional Files Tables S1 – S3. The study complied with the appropriate institutional guidelines.

**Table 2:** Demographic characteristics of patients

	EAC	BE	Without BE or EAC
Number of patients	38	18	7
M:F	34:4	18:4	2:7
Median age, yr (range)	64 (51 – 78)	56 (38 – 71)	60 (37 – 76)

### Demethylation of cell lines with 5-aza-2'-deoxycytidine

To study the effects of demethylation, triplicate cultures of the esophageal cancer cell lines OE33 and TE7 [32] were grown in RPMI 1640 supplemented with 10% foetal bovine serum at 37°C in 5% CO_2_. The OE33 cell line was established from a Barrett's associated adenocarcinoma of the lower esophagus, and the TE7 is thought to be derived from a squamous cell carcinoma of the esophagus [33]. Cells were seeded into flasks and cultured for 24 hours before they were treated with either 1 μmol/L 5-aza-2'-deoxycytidine (aza-dC, Sigma-Aldrich, Saint Louis, MO) or vehicle (0.0027% v/v final concentration acetic acid). Following a further 72 hours incubation, time for the cells to undergo at least two cycles of division [34], the medium was replaced with fresh medium not containing either aza-dC or vehicle, and the cells incubated for a further 24 hours before harvesting.

### Isolation of RNA and DNA from cell lines and patient samples

Tissues were disrupted using either disposable pestles (Edwards Instruments, Narellan, NSW, Australia) or TissueLyser with 5 mm Stainless Steel Beads (Qiagen, Hilden, Germany). RNA and DNA were isolated from cell lines and tissues from patients with EAC using Trizol (Invitrogen, Carlsbad, CA). RNA and DNA were isolated from all other biopsies using either the RNA/DNA Kit or the AllPrep DNA/RNA Mini Kit (Qiagen).

### Methylation analysis

Bisulphite modified DNA was prepared as described previously [34,35], and amplified using primer sets which did not discriminate between methylated and unmethylated sequences. The PCR primers (GeneWorks, Thebarton, SA, Australia) and conditions were specific for bisulphite modified DNA, and did not amplify unmodified DNA. All methylation analysis PCRs were performed using the QuantiTect SYBR Green PCR Kit (Qiagen) in a final volume of 15 μL, containing 1 μL of bisulphite modified DNA and a final concentration of 0.5 μmol/L of each forward and reverse primer. Bisulphite modified lymphocyte DNA, CpG methyltransferase (M.SssI) (New England Biolabs Inc., Ipswich, MA) treated lymphocyte DNA and unmodified DNA were included in each PCR run and served as unmethylated, methylated and negative controls respectively. Reactions were incubated in a Rotor-Gene 3000 (RG-3000) (Corbett Life Science, Sydney, NSW, Australia) at 95°C for 15 minutes, then 45 cycles of 95°C for 30 seconds and 60 seconds at the annealing temperature specified in Table 2, followed by a final extension of 72°C for 4 minutes. At the end of the amplification cycle the PCR products were melted by increasing the temperature from 60 to 95°C, rising 0.5 or 1°C at each step, waiting 30 seconds on the first step then 5 seconds for each step thereafter. Data was collected and analysed using the Melt Curve Analysis function of the RG-3000 Application Software v6 (Corbett Life Science) which converts the raw fluorescence data to melt curves by plotting the negative first derivative of the fluorescence with respect to temperature (-dF/dT), against temperature. The melt curve of the sample was compared to those of the unmethylated and methylated controls. A sample was considered methylated when there was a visible shift to the right of the unmethylated melt curve. The methylation extent, a function of both the number of alleles which are methylated and the density of methylation in each allele, was graded as 0 (unmethylated), 1 (low), 2 (moderate), or 3 (high methylation) according to the degree of the shift [35]. Briefly, a curve which was almost identical to the methylated control was scored as 3, a curve whose melting temperature was closer to methylated control than the unmethylated control was scored as 2, a curve which was almost identical to unmethylated control was scored 0 and the rest were scored as 1. All assessment was undertaken independently by two investigators (E.S. and P.A.D.), and if their opinions differed, consensus was reached by discussion.

### Measurement of gene expression by quantitative real-time reverse-transcription PCR

To measure gene expression, cDNA was synthesised using SuperScript II (Invitrogen) from 2 μg of RNA which had been treated with the TURBO DNA-free Kit (Ambion). Quantitative real-time reverse-transcription PCR (qRT-PCR) was performed using the QuantiTect SYBR Green PCR Kit in a final volume of 10 μL, containing 1 μL of 1/5 diluted cDNA and a final concentration of 0.5 μmol/L of each forward and reverse primer. Triplicate reactions were incubated in an RG-3000 at 95°C for 15 minutes, then 45 cycles of 95°C for 15 seconds and 45 seconds at the annealing temperature specified in Table 3, followed by a final extension of 72°C for 4 minutes. Data was collected and analysed using the RG-3000 Application Software. Threshold cycle Ct values were determined on auto-threshold settings with reference to a standard dilution curve. All mRNA quantitation data was normalised to hydroxymethylbilane synthase (HMBS) [34]. Following the PCR, the products were melted to confirm specificity, and electrophoresed on 1.5% (w/v) agarose gels stained with ethidium bromide to confirm expected product size.

**Table 3:** The primer sequences and annealing temperatures for qRT-PCR

Gene	Forward primer	Reverse primer	Annealing temperature (°C)
APC	TGGAGAACTCAAATCTTCGACA	CAATCTGTCCAGAAGAAGCCATA	62
CDKN2A	GAGGCCGATCCAGGTCAT	CCAGCGTGTCCAGGAAG	62
HMBS	ACATGCCCTGGAGAAGAATG	TTGGGTGAAAGACAACAGCA	57
ID4	CCGAGCCAGGAGCACTAGAG	CTTGGAATGACGAATGAAAACG	60
MGMT	TGGAGCTGTCTGGTTGTGAG	GCTGGTGGAAATAGGCATTC	60
RBP1	AGGCATAGATGACCGCAAG	CTCATCACCCTCGATCCAC	62
RUNX3	GCAGGCAATGACGAGAACTA	CAGTGATGGTCAGGGTGAAA	57
SFRP1	TTGAGGAGAGCACCCTAGGC	TGTGTATCTGCTGGCAACAGG	60
TIMP3	CCAGGACGCCTTCTGCAAC	CCTCCTTTACCAGCTTCTTCCC	60
TMEFF2	CAATGGGGAGAGCTACCAGA	TGGACTCCATCTCCAGATCC	62

### Statistical analyses

Statistics were performed using GraphPad InStat version 3.0a or Prism version 5.0a for Macintosh (GraphPad Software, San Diego California USA, ). Gene expression in cell lines treated with either aza-dC or vehicle was compared using Student's t-test. Methylation frequency in tissues was compared using Fisher's exact test. The number of methylated genes in a specimen and gene expression was compared using Kruskal-Wallis with Dunn's post-test. Methylation extent and gene expression in unmethylated and methylated tissues was compared using the Mann-Whitney test. All statistics were considered significant when the two tailed P ≤ 0.05.

## Conclusion

We have measured methylation of genes in BE and EAC and found significant methylation in metaplastic BE, which for seven of the nine genes studied did not differ in frequency from that found in EAC. This study provides important confirmatory evidence to support the concept that BE is a highly abnormal tissue, more similar to cancer tissue than to normal squamous epithelium.

## Competing interests

The authors declare that they have no competing interests.

## Additional files

**Table 4:** Table S1 – Demographic details of patients with EAC.

Patient	Gender	Age(yr)	Differentiation^a^	S-EAC^b^	D-EAC^b^	EAC^b^
0226	M	53	P	+	-	+
0227	M	58	M	+	+	-
0230	M	77	M-P	+	+	+
0235	M	61	M	+	-	+
0246	F	62	W	+	+	+
0250	M	68	M-P	+	+	+
0253	M	77	M-P	+	-	+
0292	M	55	M	+	-	+
0306	M	68	P	+	-	+
0307	M	58	M	+	-	+
0316	M	69	P	+	+	+
0329	M	67	P	-	-	+
0339	F	63	P	+	-	+
0343	F	62	P	-	-	+
0347	M	56	P	+	-	+
0355	F	62	P	+	-	+
0369	M	75	M	+	-	+
0374	M	65	M	+	-	+
0375	M	72	M	+	-	+
0380	M	57	M	+	-	+
0385	M	70	M	+	-	+
0406	F	53	M	+	-	+
0410	M	62	M	+	-	+
0415	M	56	P	-	+	+
0417	M	73	P	-	+	+
40320	M	61	M	-	-	+
40323	M	60	P	-	-	+
40325	M	61	M-P	-	-	+
40328	M	65	M	-	-	+
40331	M	64	P	-	-	+
40337	M	70	P	-	-	+
40338	M	71	W	-	-	+
40341	M	78	P	-	-	+
40345	M	60	M	-	-	+
40355	M	65	M-P	-	-	+
40357	M	65	M-P	-	-	+
53145	M	51	M	-	-	+
54017	M	76	P	-	-	+

^a^W, well; M, moderate; P, poor

^b^+ tissue available; – no tissue available

**Table 5:** Table S2 – Demographic details of patients with BE

Patient No.	Gender	Age (yr)	S-BE^a^	BE^a^	No. of BE biopsies
1	M	71	+	+	2
2	M	51	+	+	3
3	F	70	+	+	2
4	M	65	+	+	2
5	M	61	+	+	2
6	M	43	+	+	2
7	M	38	+	+	5
8	M	54	+	+	1
9	M	53	-	+	1
10	M	70	+	+	2
11	M	64	+	+	3
12	M	68	+	+	2
13	F	54	-	+	2
14	M	52	+	+	3
15	F	50	+	+	2
16	M	49	+	+	2
17	M	58	+	+	2
18	F	58	+	+	2

^a^+ tissue available; – no tissue available

**Table 6:** Table S3 – Demographic details of patients without BE or EAC

Patient No.	Gender	Age (yr)	No. of biopsies
1	M	49	3
2	M	76	3
3	F	66	3
4	M	37	3
5	M	37	2
6	F	60	3
7	M	69	2
